# Supplementary material for: Hyperuniformity and phase enrichment in vortex and rotor assemblies
Source: Nat Commun. 2022 Feb 10;13:804. doi: 10.1038/s41467-022-28375-9 (PMC8831603; doi:10.1038/s41467-022-28375-9)
Supplement: Supplementary file 1 — Supplementary Information [file 41467_2022_28375_MOESM1_ESM.pdf]

Supplementary Information — Hyperuniformity and phase enrichment in vortex and rotor assemblies

Naomi Oppenheimer,[1,*] David B. Stein,[2] Matan Yah Ben Zion,[3] and Michael J. Shelley[2,4,†]

[1]*School of Physics, and the Center for Physics and Chemistry of Living Systems, Tel Aviv University, Tel Aviv 6997801, Israel*

[2]*Center for Computational Biology, Flatiron Institute, New York, NY 10010, USA*

[3]*Laboratoire Gulliver, UMR CNRS 7083, ESPCI Paris, PSL Research University, 75005 Paris, France*

[4]*Courant Institute, New York University, New York, NY 10012, USA*

Rate of convergence to hyperuniformity

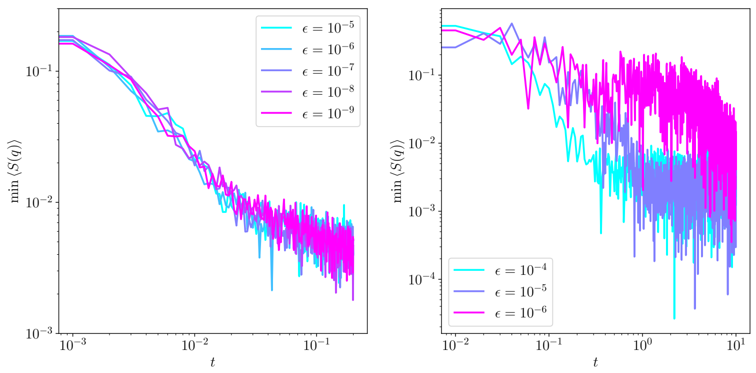

Supplementary Figure 1. The value of $S(q_{min})$ as a function of time, where q_{min} is inversely proportional to the window size. Simulations were done on a system of 10,000 particles. On the left results are shown for a BDD system with steric interaction, showing that convergence to hyperuniformity is independent of step-size, on the right for point vortices, as the tolerance is reduced (i.e. the average step size is decreased) the ensemble takes longer to reach hyperuniformity.

* naomiop@gmail.com

† mshelley@flatironinstitute.org

Symplectic integration

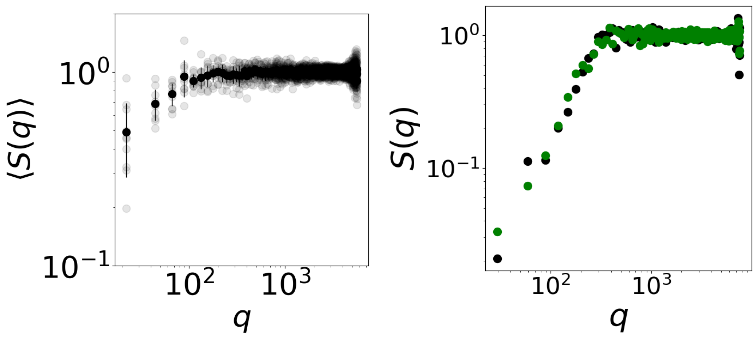

Supplementary Figure 2. (left) We have tested a symplectic integration scheme, following Zhang and Qin (Computers Math. Applic., 1993). The scheme is much more costly and we were able to test a small ensemble of 1,000 point vortices over $\sim 10^5$ cycles. The structure factor shows little to no signs of hyperuniformity. The Hamiltonian is conserved to within a relative error of 10^{-4} and the second moment is conserved to within 10^{-11}. (right) Running the same symplectic integration scheme but starting with a hyperuniform arrangement, the ensemble stays hyperuniform. Here in a simulation of 1,000 point vortices. Green is the initial configuration and black is the result after $\Delta t \sim 5 \times 10^4$ cycles. As noted in the main text, we suspect that perturbations that break the rotational symmetry of the Hamiltonian are necessary for it to overcome a dynamical bottleneck and reach a hyperuniform state.

Global shearing flow

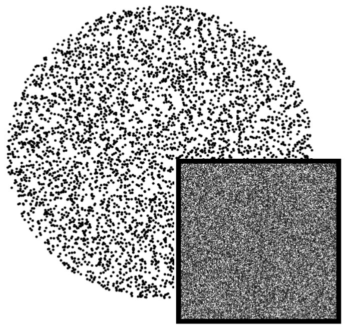

Supplementary Figure 3. A global shear flow which increases as the distance from the center squared, $\mathbf{v} = r^2 \hat{r}$, was applied on a uniform distribution of 5,000 particles with only steric interactions. Snapshot taken after 50,000 timesteps. No hyperuniformity is observed as is clear from the structure factor which is shown on top.

Average relative deviation

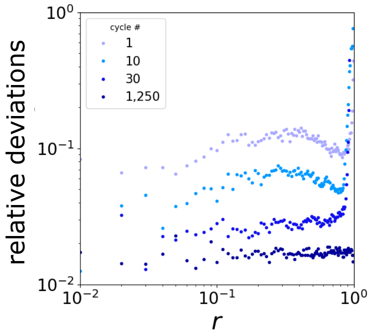

Supplementary Figure 4. An azimuthal average of the relative deviation, as defined in the main text for different cycles showing edge current at initial times which relaxes at a later time.

Hyperuniformity in surface rotors (the quasigeostrophic limit) and rotors near a substrate

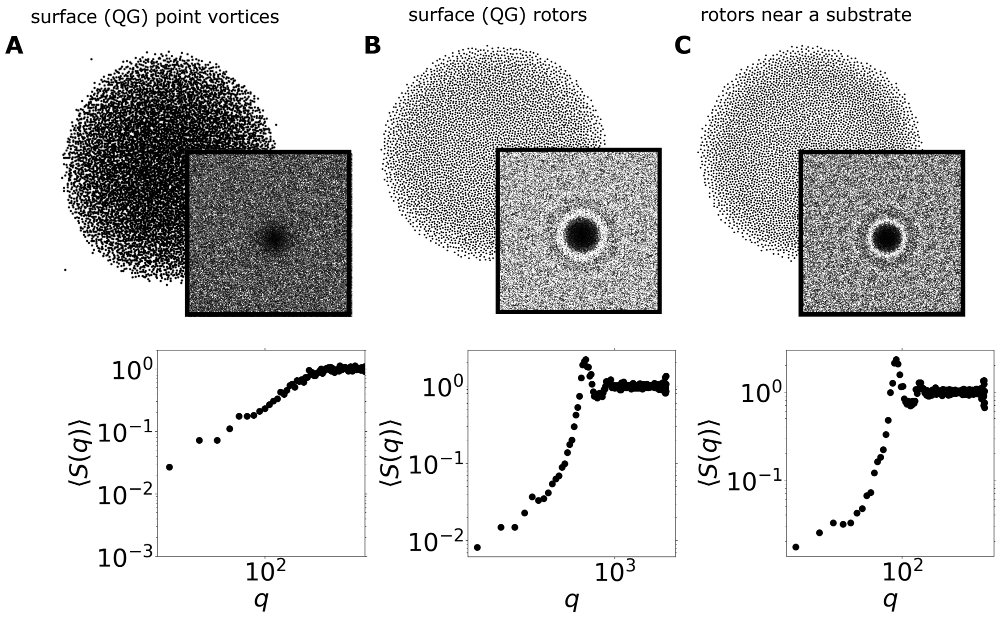

Supplementary Figure 5. Hyperuniformity in other rotor systems. a) point vortices near the surface of a fluid, which scale as $1/r^2$, equivalent to the quasigeostrophic (QG) limit or the large SD limit of a membrane. b) surface rotors - that is a $1/r^2$ flow, including steric interactions. c) a system of rotors near a solid substrate, which scale as $\sim 1/r^3$. All cases are hyperuniform as can be seen from the structure factor shown in the inset, and its angular average plotted below. Simulations for (a) were done for 10,000 vortices in an inital radius of 1. Simulations for (b) and (c) were done for 5,000 particles, with soft core steric interactions, and an area fraction of 0.3.

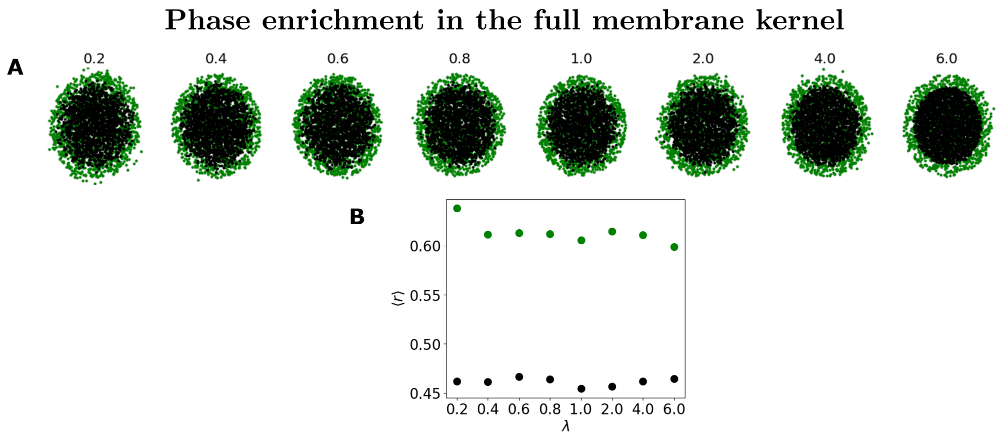

Supplementary Figure 6. Results for the phase enrichment of the full membrane kernel. Two populations of vortices — half (2500) with a high circulation Γ_h (in black) the other half with circulation Γ_l (in green) such that the ratio is $\Gamma_h/\Gamma_l = 128$. a) vortex configurations for different values of the Saffman-Delbruck length λ as stated above the plot, at small λ the system is at the quasigeostrophic limit scaling as $1/r^2$, at the opposite limit of large λ we are in the limit of point vortices in an ideal Euler fluid where the velocity scales as $1/r$. b) the average inner and out radii do not show a strong dependence on the SD length.

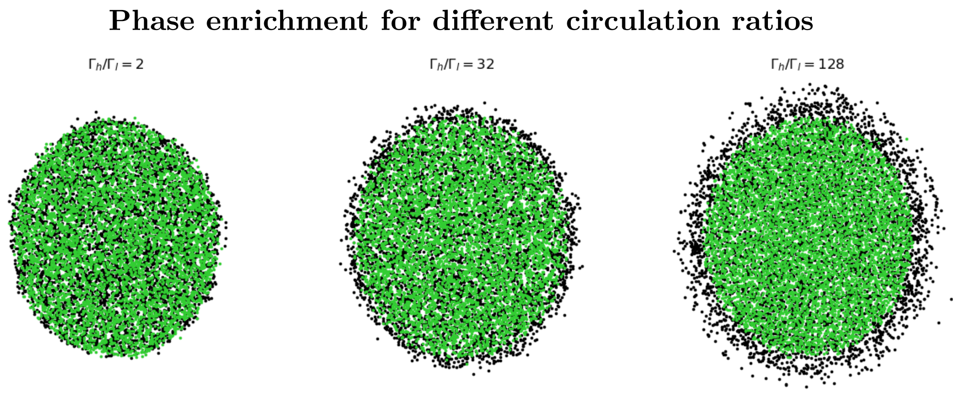

Supplementary Figure 7. Phase enrichment in an ideal fluid. Simulation results for 5,000 point vortices of low circulation, Γ_l and 5,000 of high circulation, Γ_h. Outer radius increases with the circulation ratio, consistent with the simple heuristic model presented below.

Supplementary Note 1: A simplified model of two populations

In here we show that when there are two populations — fast and slow vortices — a steady state is possible in which each population has a different radii. We start with two continuous populations of rotors, ρ_h of rotors with a high circulation Γ_h, and ρ_l that has a low value of circulation Γ_l. The two are initially distributed uniformly within a disk of radius R, such that $\rho_h(t=0) = \rho_l(t=0) = N/\pi R^2$, where N is the number of vortices in each population. We assume that there is a steady state. We further assume that at that steady state the two populations are still uniform but with a different radii r_h and r_l, such that $\rho_h(ss) = N/\pi R_h^2$, and $\rho_l(ss) = N/\pi R_l^2$, where ss stands for steady state. This last assumption is a considerable simplification, as the simulations indicate that ρ_l is not uniform at steady state, but decays close to the edge. With these assumptions in mind, we can use the two conservation laws, of the Hamiltonian and of the second moment, in order to find r_h and r_l. From conservation of the second moment we have,

$$M = (\Gamma_h + \Gamma_l)\frac{NR^2}{2} = \Gamma_h \frac{NR_h^2}{2} + \Gamma_l \frac{NR_l^2}{2}, \tag{1}$$

where in Eq. 1 we used that for a uniform distribution in a disk or radius R, the second moment is given by

$$M = \int \Gamma \rho(r) r^2 r dr d\theta = \frac{\Gamma N R^2}{2}. \tag{2}$$

The Hamiltonian for two populations is given by,

$$\mathcal{H} = -\frac{1}{2}\int\int \Psi(|\mathbf{r}-\mathbf{r}'|)\omega(\mathbf{r})\omega(\mathbf{r}')\mathbf{drdr'} \quad ; \quad \omega = \mathbf{\Gamma_h}\rho_\mathbf{h}(\mathbf{r}) + \mathbf{\Gamma_l}\rho_\mathbf{l}(\mathbf{r}). \tag{3}$$

For point vortices $\Psi = \frac{1}{2\pi}\log|\mathbf{r}-\mathbf{r}'|$. We calculate this integral initially with $\rho_h = \rho_l = \frac{N}{\pi R^2}$, to get

$$\mathcal{H} = -\frac{N^2(\Gamma_l + \Gamma_h)^2}{8}(4\log R - 7). \tag{4}$$

At steady state a bit of care must be taken with the integration limits. We assume that the high circulation vortices are concentrated within a radius $R_h < R_l$, such that at distances $R_h < r < R_l$ only ρ_l exists. We get that at steady state

$$\begin{aligned}\mathcal{H} =& -\frac{N^2\Gamma_h^2}{8}(4\log R_h - 7) - \frac{N^2\Gamma_l^2}{8}(4\log R_l - 7) - 4\frac{\Gamma_h\Gamma_l N^2}{R_l^2 R_h^2}\left\{\frac{R_h^4}{16}(4\log R_h - 7) + \right.\\ &+ \frac{1}{48}\left[(R_l - R_h)(3R_l^3 + 13R_l^2 R_h + 16 R_l R_h^2 + 10R_h^3) - 6R_l^4\log(R_l)\right.\\ &\left.\left. -6(R_l^2 - R_h^2)^2\log(R_l - R_h) + 6R_h^4\log(R_h)\right]\right\}.\end{aligned} \tag{5}$$

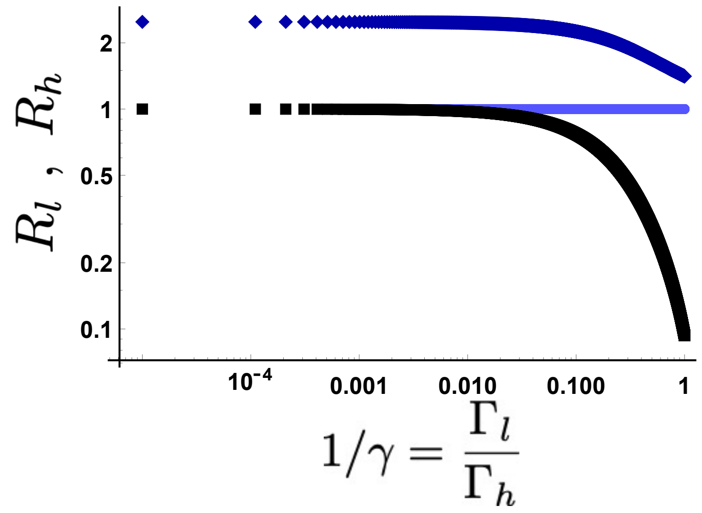

Supplementary Figure 8. The analytic solution for the possible radii of two uniform distribution, high (low) circulation result shown in black (dark blue), light blue gives the second possible solution where the radii stays the same and equal to the initial radius. As can be seen, at high circulation ratios (large γ, the radii asymptote to a constant.

We can express R_h from Eq. 1, and compare Eq. 4 and Eq. 5 to solve for R_l. Numerically, we get two solutions for all values of $\gamma = \Gamma_h/\Gamma_l$, one solution in which the initial radius, R is maintained for the two populations, a second solution gives a lower value for R_h and a higher value for R_l. These solutions are plotted in Fig. 8. The asymptotic limits for high circulation ratios are given in the main text.
